# Development and pilot evaluation of a personalized decision support intervention for low risk prostate cancer patients

**DOI:** 10.1002/cam4.2685

**Published:** 2019-11-12

**Authors:** Jeffrey Belkora, June M. Chan, Matthew R. Cooperberg, John Neuhaus, Lauren Stupar, Tia Weinberg, Jeanette M. Broering, Imelda Tenggara, Janet E. Cowan, Stan Rosenfeld, Stacey A. Kenfield, Erin L. Van Blarigan, Jeffry P. Simko, John Witte, Peter R. Carroll

**Affiliations:** ^1^ Institute for Health Policy Studies University of California San Francisco CA USA; ^2^ Department of Urology University of California San Francisco CA USA; ^3^ Department of Epidemiology and Biostatistics University of California San Francisco CA USA; ^4^ Department of Pathology University of California San Francisco CA USA

**Keywords:** behavioral science, cancer education, ethical considerations, prostate cancer

## Abstract

**Objectives:**

Development and pilot evaluation of a personalized decision support intervention to help men with early‐stage prostate cancer choose among active surveillance, surgery, and radiation.

**Methods:**

We developed a decision aid featuring long‐term survival and side effects data, based on focus group input and stakeholder endorsement. We trained premedical students to administer the intervention to newly diagnosed men with low‐risk prostate cancer seen at the University of California, San Francisco. Before the intervention, and after the consultation with a urologist, we administered the Decision Quality Instrument for Prostate Cancer (DQI‐PC). We hypothesized increases in two knowledge items from the DQI‐PC: How many men diagnosed with early‐stage prostate cancer will eventually die of prostate cancer? How much would waiting 3 months to make a treatment decision affect chances of survival? Correct answers were: “Most will die of something else” and “A little or not at all.”

**Results:**

The development phase involved 6 patients, 1 family member, 2 physicians, and 5 other health care providers. In our pilot test, 57 men consented, and 44 received the decision support intervention and completed knowledge surveys at both timepoints. Regarding the two knowledge items of interest, before the intervention, 35/56 (63%) answered both correctly, compared to 36/44 (82%) after the medical consultation (*P* = .04 by chi‐square test).

**Conclusions:**

The intervention was associated with increased patient knowledge. Data from this pilot have guided the development of a larger scale randomized clinical trial to improve decision quality in men with prostate cancer being treated in community settings.

## Graphical abstract

Men with low‐risk prostate cancer are at risk for making decisions based on misconceptions about treatment options and outcomes. A decision support intervention, delivered by premedical students acting as health coaches, increased patient knowledge of key facts about prognosis.

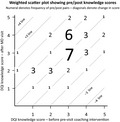

## 1 BACKGROUND

Patients with low‐risk prostate cancer are vulnerable to making decisions based on incomplete information.^1^ Patients have misconceptions about the risks and benefits of surgery, radiation, and active surveillance.^2^, ^3^, ^4^ This can result in prostate cancer survivors feeling that they had more or less treatment than they would have chosen if they had been fully informed and more involved in their decisions.^5^, ^6^, ^7^, ^8^

A systematic review with meta‐analysis of randomized controlled trials concluded that decision aids are associated with increases in patient knowledge,^9^ among other benefits. Communication aids include question‐listing interventions.^10^ A systematic review with meta‐analysis found that these are associated with increased involvement in the form of question‐asking.^11^ Members of our team have developed communication aiding interventions showing psycho‐social benefits for men with prostate cancer, including increases in decision self‐efficacy (DSE) and reductions in decisional conflict and regret.^12^

It appears decision and communication aids can address deficits in patients being informed and involved. However, we identified two gaps in the literature.

First, decision aids in prostate cancer have not yet provided personalized estimates of risk and benefit. Two randomized controlled studies of decision aids in prostate cancer found increased knowledge.^13^, ^14^ However, these decision aids were not targeted specifically at low‐risk prostate cancer using personalized estimates of risk.^15^ Greater personalization of decision aids for low‐risk patients is now possible because researchers are beginning to report long‐term outcomes data about mortality and side effects while stratifying results according to risk level.^16^, ^17^

The second gap in the literature is that researchers have not yet studied the delivery of decision and communication aids by students as part of their pre‐medical training. A recent review of evidence^18^ found three studies, in domains other than prostate cancer, where professional health coaches delivered decision aids that were associated with increased patient knowledge.^19^, ^20^, ^21^ Subsequent to the publication of this review, one study by Mishel et al in prostate cancer found a strong effect on knowledge when nurses coached patients in the use of decision and communication aids.^14^

Addressing these gaps would contribute important knowledge about the impact of more specific patient education; and whether delivery of patient education interventions can be task‐shifted to students.

To address these gaps, our team developed a multi‐component decision support intervention. First, we asked whether an intervention delivered by pre‐medical student interns would be acceptable to a focus group of stakeholders. Second, we asked if a personalized decision support intervention was associated with improved patient knowledge about early‐stage prostate cancer.

## 2 METHODS

### 2.1 Approach and study design

We approached this research as formative work to assess the acceptability and efficacy of a novel decision support intervention, while generating pilot data to estimate effect sizes for a future randomized controlled trial. We developed our intervention using qualitative methods and conducted a pre/post test with patients at our academic medical center. We obtained ethics approval (14‐13332) from the Committee on Human Research at the University of California, San Francisco (UCSF). We registered the study on http://www.ClinicalTrials.gov as number NCT02451345**.**

### 2.2 Target population and study samples

Our target population was men with low‐risk prostate cancer being treated in academic and community settings in the United States. For the intervention design phase, we convened a sample of 8 patients, 1 family member/caregiver, and 7 healthcare professionals. For the intervention testing phase, we approached patients diagnosed with low‐risk disease at the University of California, San Francisco to discuss treatment options with a urologist. The inclusion criteria included men age ≥18, who could speak and read English, and with newly diagnosed (within 6 months), low‐risk prostate cancer, who have not yet received therapy. Low‐risk was defined as: Gleason score ≤3+4, stage ≤T2N0M0, PSA ≤10 ng/mL.

### 2.3 Outcomes, measures, and instruments

#### 2.3.1 Intervention design phase

We used a survey instrument from the International Patient Decision Aids Standards (IPDAS) to measure the stakeholder endorsement of our decision aid.^22^ We limited our questionnaire to 12 questions in the qualifiying and certifying criteria. See online supplemental materials Data S1.

We also asked stakeholders to rate the acceptability of our coaching intervention using the Decision Support Assessment Tool,^23^ a written survey instrument designed to evaluated the provision of decision coaching. See online supplemental materials Data S1.

#### 2.3.2 Intervention testing phase

For the intervention testing phase, we collected patient demographics at baseline, and measured decision self‐efficacy immediately before and immediately after the intervention. Our intention was to orient patients to their treatment options and outcomes using the decision aid before the urologist consultation. We also wanted to help them list questions. We hypothesized that the decision aid and question listing would increase patient decision self‐efficacy. We measured patient knowledge, as described below, before the intervention and after the medical consultation. We wanted the patient to ask questions and emerge from the consultation with increased knowledge.

##### Decision quality instrument with knowledge subscale

The Decision Quality Instrument‐Early Prostate Cancer Treatment has a knowledge subscale with 11 questions which can be provided to patients in the form of a multiple‐choice quiz.^24^ We chose five questions about survival outcomes and side effects that were addressed by our decision aid. (See Table 1). We hypothesized that we would see a pre/post increase in the proportion of patients who answered the first two knowledge items correctly: How many men diagnosed with early‐stage prostate cancer will eventually die of prostate cancer? How much would waiting 3 months to make a treatment decision affect chances of survival? Correct answers were: “Most will die of something else” and “A little or not at all.” These items are most relevant to patient understanding that their condition is not urgently life‐threatening, and there is time to weigh all options thoroughly.

**Table 1:** Survey to assess patient knowledge

Five items from decision quality instrument‐early prostate cancer treatment^24^
1. Without treatment, about how many men diagnosed with early‐stage prostate cancer will eventually die of prostate cancer? Responses: Most will die of prostate cancer; About half will die of prostate cancer; Most will die of something else*.
2. For most men with early‐stage prostate cancer, how much would waiting a few months to make a treatment decision hurt their chances of survival? Responses: A lot; Somewhat; A little or not at all*.
3. In the first few years after treatment for prostate cancer, which is more likely to cause bowel problems? Responses: Surgery; Radiation*; Both surgery and radiation are equally likely to cause bowel problems.
4. In the first few years after treatment for prostate cancer, which is more likely to cause sexual problems with erections? Surgery*; Radiation; Both surgery and radiation are equally likely to cause sexual problems.
5. In the first few years after treatment for prostate cancer, which is more likely to cause dripping or leaking urine? Responses: Surgery*; Radiation; Both surgery and radiation are equally likely to cause dripping or leaking urine.

* denotes the correct answer.

##### Decision self‐efficacy item

To assess decision self‐efficacy, we used an item from the decision self‐efficacy scale.^25^ This item was sensitive to our question‐listing intervention in a prior randomized controlled trial.^12^ The item requested a 0‐4 confidence rating that I can "Figure out the treatment choices that best suit me."

### 2.4 Data collection procedures

#### 2.4.1 Intervention design phase

Decision scientists on our team designed an initial prototype of our decision aid, using the SCOPED model as a conceptual framework.^26^ SCOPED is an acronym whose letters represent steps in reflecting critically on a decision: Situation, Choices, Objectives, People, Evaluation, and Decisions.

To refine this prototype, we identified a representative group of stakeholders. We conducted rounds of feedback until all the stakeholders endorsed the decision aid according to the IPDASi standards described above.

In order to systematically incorporate stakeholder feedback, we used the Nominal Group Technique.^27^ The Nominal Group Technique is a focus group technique that captures stakeholder input in writing first, to prevent dominance of any especially verbal members of the group. In each round of feedback, we surveyed the stakeholders about the current version of the decision aid; discussed their survey responses; and then voted on the acceptability of the decision aid. We continued making changes to the decision aid and getting feedback until all stakeholders rated the decision aid as acceptable.

Our coaching intervention to deliver the decision aid was based on an established approach to question listing, described in the literature as Consultation Planning.^26^ To adapt Consultation Planning for this intervention, we recorded all coaching sessions, and reviewed recordings with the stakeholder team during our biweekly calls. We asked stakeholders to rate the coaching process using the Decision Support Analysis Tool (DSAT) survey, capturing suggestions for improvement and repeating until we arrived at consensus endorsement of the intervention design.

#### 2.4.2 Intervention testing phase

##### Study sample

We enrolled a convenience sample of 51 patients seeing 7 urologists at UCSF, between 4/1/2015 and 2/7/17. Part‐time study coordinators approached these patients based on the coordinator's availability and overlap with their urologist's schedule. After enrollment, student coaches contacted the patients to administer the intervention by telephone and survey instruments by email.

### 2.5 Analysis plan

#### 2.5.1 Intervention design phase

We documented the ongoing suggestions for improvement from stakeholders. All suggested modifications were considered by study personnel (including investigators, software developers, and patient representatives). The study's co‐investigators weighed additional factors such as cost and technical feasibility in incorporating panelist feedback.

#### 2.5.2 Intervention testing phase

Decision self‐efficacy: We graphed the distribution of DSE scores before compared to after the intervention, and counted the number and proportion of patients whose DSE score rose vs fell. We also compared the mean DSE before and after the intervention using a paired *t* test.

Decision Quality Instrument—Knowledge: We graphed the distribution of knowledge scores before compared to after the intervention, and counted the number and proportion of patients whose knowledge scores rose vs fell. We compared the mean knowledge score before and after the intervention using a paired *t* test. We computed the proportion of patients who answered the first two knowledge items correctly. Then, we used McNemar's test for the hypothesis of no difference in number of patients who answered both correctly before compared to after the intervention. We used Release 10 of Stata Statistical Software for all statistical analyses.^28^

## 3 RESULTS

### 3.1 Intervention design phase

The stakeholder team arrived at a consensus endorsement of our initial decision aid after three rounds of feedback, and a consensus endorsement of our coaching intervention after one round. Based on this feedback, we concluded the decision aid and coaching intervention were feasible and acceptable for inclusion in the intervention testing phase of the study. We trained our existing premedical student interns to deliver the intervention.^29^

The training for this intervention was based on a question‐listing curriculum we have implemented since 2012 with the premedical student intern workforce at UCSF. These student interns participate in a service learning program known as the Patient Support Corps. Through the Patient Support Corps, the students earn academic credit while gaining experience working as health coaches in our medical center. Each year, we recruit our interns from the undergraduate student population at the University of California, Berkeley. We select students after a screening process that includes a written application and interview. The application and interview focus on the student's competence in neutral, non‐directive coaching.

Through this screening process, we identify students who are skilled at gathering information from others; who can summarize and paraphrase information in a neutral fashion; and who will escalate problems to supervisors when situations arise outside of their scope. Then the Director of the Patient Support Corps (first author JB), along with the program coordinator (author TW), train the students in their specific question‐listing tasks.

We administer 16 hours of classroom training in which the students learn a process for eliciting and documenting patient questions, known as the SLCT process.^29^ After reviewing videos of the process in action, trainees role‐play in pairs and the instructors review recordings of their role‐plays and provide feedback. Then the trainees are paired up with experienced student interns, who shadow them during patient interactions until the trainees are ready to interact with patients alone.

After the trainees begin interacting with patients alone, they submit recordings of their interactions to the program director and coordinator, who review recordings in group meetings every week, and provide ongoing training and quality improvement.

#### 3.1.1 Coaching and question‐listing process

We were able to leverage our existing training for coaches because the final study coaching process closely resembled our existing question‐listing intervention, described in the literature.^26^ The only material difference in this project was that, in addition to open‐ended question prompts, the coach also used the decision aid content as additional question prompts. The coach did this by reviewing the decision aid with the patient one screen at a time, checking for questions, then writing the questions down and asking for elaboration. Also, based on focus group feedback, we asked the coaches to check for patient understanding and direct the patient to additional help text in the decision aid when something was unclear.

#### 3.1.2 Decision aid content and interface

Our software team coded four versions of our decision aid as a result of the iterative feedback we collected. Readers may request a copy of the decision aid in portable document format from the corresponding author. Our software development team deployed this decision aid as a web application using a JavaEE web profile and ran it on Amazon Elastic Beanstalk to provide automatic updates and resiliency. The application stored and retrieved data from a database configured using the Research Electronic Data Capture platform (REDCap).^30^

### 3.2 Intervention testing phase

#### 3.2.1 Sample description

Between April 2015 and February 2017 (4/1/15‐2/7/17), our clinic research coordinators enrolled a convenience sample of 51 men seeing 7 urologists in our clinic. The men had a median age of 63 (mean 62). Racial and ethnic representation was 43 Caucasian/White, 2 Asian/Pacific, 6 Other/Mixed/ Unknown including 4 Hispanic/Latino. For employment status, 21 reported an employment status of Working, 14 others Self‐employed, 11 Retired, and 5 Other/Unknown. Cancer of the Prostate Risk Assessment (UCSF‐CAPRA) scores ranged from 1‐6 (median: 2). T1c was the most common stage (63%).

#### 3.2.2 Decision self‐efficacy

For decision self‐efficacy (0‐4 confidence rating that I can "Figure out the treatment choices that best suit me"), the distribution of scores was similar before compared to after the intervention. The scale showed a ceiling effect, as the most frequent score before and after was the highest score, 4. The pre and post‐intervention means were not statistically significantly different (3.43 to 3.47, *P* = .62).

**Figure 1:**
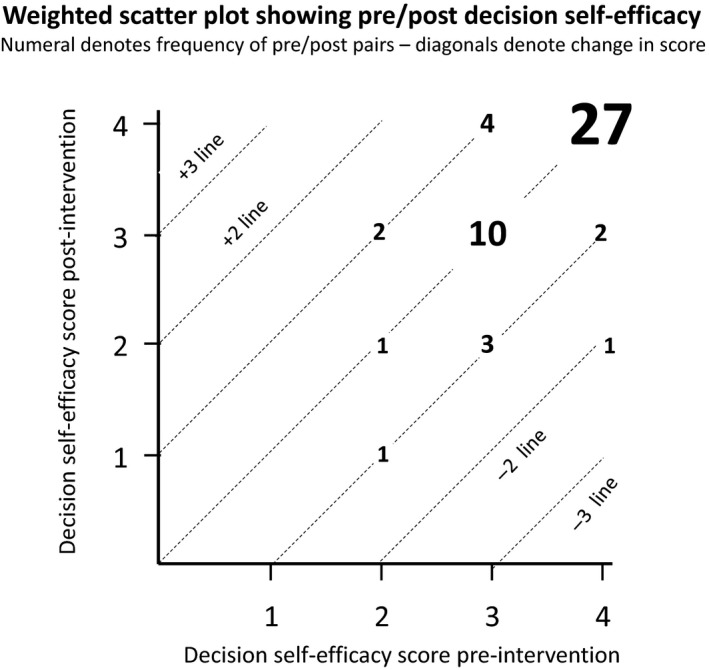
Scatterplot of paired decision self‐efficacy scores (before and after)

We graphed the joint distribution of ratings, pre and post (see Figure 1). Figure 1 shows parallel 45‐degree lines corresponding to changes in score of −2, −1, 0, and +1, while the responses are superimposed on the lines in bubbles whose size reflects the frequency of each pre/post combination. For example, a bubble on the +1 line shows 2 respondents rated their self‐efficacy at 2 before and 3 after the intervention.

Overall, Of 51 respondents to the DSE pre and post‐intervention, 38 scores (72%) stayed the same (shown on the 0‐change line), with 27 (51%) holding perfect at 4/4. Six scores (11%) went up one point, while six (11%) went down 1 point and one (2%) went down 2 points.

#### 3.2.3 Decision quality instrument—knowledge

For knowledge, the distribution of total knowledge score after was shifted upwards compared to before. The raw improvement in means (2.84‐3.16) was not statistically significant (*P* = .16).

**Figure 2:**
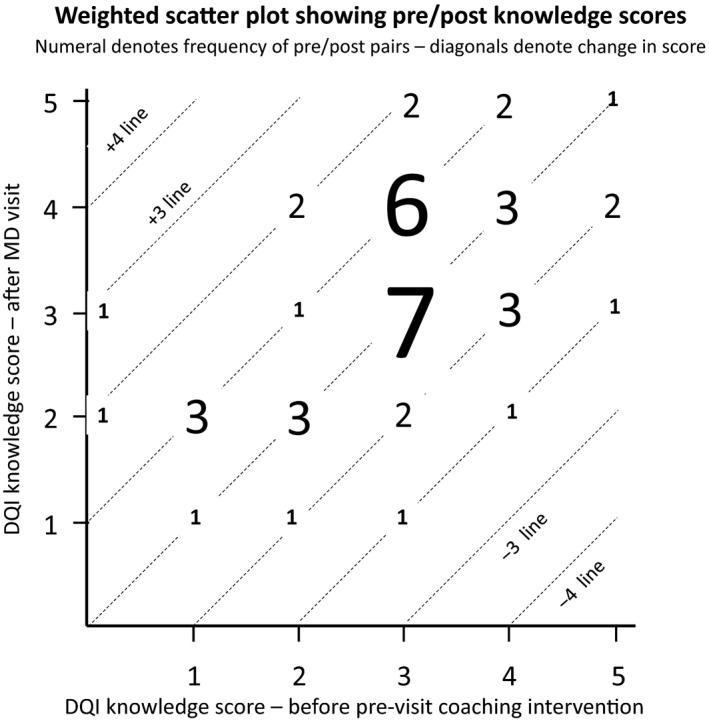
Scatterplot of paired total knowledge scores (before intervention and after consultation)

We graphed the joint distribution of knowledge scores (see Figure 2). Figure 2 shows parallel 45 degree lines corresponding to changes in score of −2, −1, 0, +1, +2, and +3, while the responses are superimposed on the lines in bubbles whose size reflects the frequency of each pre/post combination. For example, a bubble on the +1 line shows 3 respondents answered 1 item correctly before and 2 items correctly after the intervention.

Overall, Figure 2 reveals 15 scores (29%) staying flat, 12 (27%) going up one point, 5 (11%) going up two points, and one (2%) going up 3 points; while 8 (18%) went down 1 point, and 3 (7%) went down 2 points. There were 18 (41%) scores that went up, and 11 (25%) that went down. This raw difference was not a statistically significant difference (binomial sign test *P* = .13).

We had previously identified the first two items of the DQI knowledge survey as most relevant to our decision support intervention. Before the intervention, 35/56 (63%) got both these questions right. After the consultation, 36/44 (82%) got both these questions right (*P* = .04 by Chi‐Square test).

Among respondents who answered the questions at both timepoints, the number moving from at least one incorrect to both correct (7, or 16%) was higher than the number moving from both correct to at least one incorrect (1, or 2%; McNemar *P* = .08). See Table 2.

**Table 2:** Patient performance on first two knowledge items before and after intervention

Before Intervention	After Intervention	
	At least 1 incorrect	Both correct
At least one incorrect	7	7
Both correct	1	29

## 4 DISCUSSION

We designed and tested a multi‐component intervention with personalized decision and communication aids that premedical students could deliver by telephone. Our decision aid broke new ground by incorporating long‐term, patient‐specific, and personalized data on both survival and side effects. We adapted a prior communication aid (Consultation Planning) and prompted patient questions in categories corresponding to the six decision aid topics. The close integration of a personalized decision aid with coached question‐listing is also novel.

We were surprised that the intervention was not associated with pre/post changes in decision self‐efficacy. A recent study of prostate cancer patients in the UK found that Consultation Planning alone was associated with a significant pre/post change in DSE.^12^ As opposed to that study, our population demonstrated a ceiling effect that left little room for improvement. We believe that patients in community settings may demonstrate lower self‐efficacy levels and benefit more from the intervention.

The direction and magnitude of improvement in knowledge was encouraging. A subset of two key knowledge questions were especially sensitive to our intervention. We will use these two items as the primary outcome in a randomized controlled trial of our intervention vs usual care in community settings, with decision self‐efficacy as a secondary outcome.

### 4.1 Study strengths

The strengths of our study included the participation of diverse stakeholders, notably patient representatives, during the design phase. We designed an innovative multi‐component intervention that was delivered by members of an untapped workforce—premedical students who earned academic credit while serving as health coaches. The intervention broke new ground in prostate cancer education by personalizing our decision aid with risk information based on each patient's clinical characteristics. In addition, we integrated the decision aid with our coach‐led question‐listing intervention, to assure that each component of our intervention flowed smoothly into the next.

### 4.2 Study limitations

Our formative study recruited a convenience sample of patients in an academic medical center, had no control group, and relied on self‐reported measures collected before and after the intervention (self‐efficacy) and consultation with a urologist (knowledge). We observed knowledge gains but in the absence of a control group, we do not know if they would have occurred even without the intervention. Other limitations include sampling bias (we invited patients at convenient times for the study coordinator), motivational bias (patients who consented may be different than those who did not), agreement bias (patients may have wanted to please study personnel or the clinical care team with their answers), and maturation bias (there could have been changes in the environment over time relevant to our study outcomes). One of our measures exhibited a strong ceiling effect, which may not be as evident in other settings. This study was conducted in an academic center with urologists who are highly specialized in the care of patients with low‐risk prostate cancer, which may not be representative of all prostate cancer care settings.

### 4.3 Clinical implications

The ceiling effect in decision self‐efficacy is surprising when juxtaposed with relatively low knowledge scores. The mode of the DSE distribution was the maximum score (4/4), while the mode of the total knowledge score was 60% (3/5). This suggests that patient confidence about making decisions exceeded patient knowledge. Our finding suggests that patients may need more education than they report, if they are to make decisions based on valid information. Therefore, in order to assure truly informed consent, clinicians should check explicitly for understanding on key facts, whether or not the patient asserts self‐efficacy for decision making. In the case of early‐stage prostate cancer, two key misconceptions include how many men diagnosed with early‐stage prostate cancer will eventually die of prostate cancer (most will die of something else); and how much would waiting 3 months to make a treatment decision affect chances of survival (a little or not at all). These items are most relevant to patient understanding that their condition is not urgently life‐threatening, and there is time to weigh all options thoroughly.

## 5 CONCLUSIONS

Our multi‐component decision aid intervention has potential for reducing knowledge deficits about early‐stage prostate cancer. We would like to further examine whether the intervention will improve knowledge and decision self‐efficacy, in a population with lower decision self‐efficacy than seen in our sample. To more definitively address these questions we have designed and are implementing a cluster‐randomized controlled trial with sites in community settings.

## CONFLICT OF INTEREST

The authors have no conflict of interest to declare.

## Data Availability

The data that support the findings of this study are available from the corresponding author upon reasonable request.
