# Supplementary material for: Development and pilot evaluation of a personalized decision support intervention for low risk prostate cancer patients
Source: Cancer Med. 2019 Nov 12;9(1):125–32. doi: 10.1002/cam4.2685 (PMC6943165; doi:10.1002/cam4.2685)
Supplement: Supplementary file 1 [file CAM4-9-125-s001.docx]

***Supplemental materials***

1. **Checklist to Assess Acceptability of Patient Decision Aid**

**Twelve items from IPDASi**^[21](#_ENREF_21)^ **Checklist**

Qualifying criteria with Yes/No response options:

The patient decision aid…

1. Describes the health condition or problem (treatment, procedure, or investigation) for which the decision is required
2. Explicitly states the decision that needs to be considered
3. Describes the options available for the decision
4. Describes the positive features (benefits or advantages) of each option
5. Describes the negative features (harms, side effects, or disadvantages) of each option
6. Describes what it is like to experience the consequences of the options (e.g. – physical, phychological, social)

Certifying criteria with 4 response options: Strongly Agree, Agree, Disagree, Strongly Disagree

The patient decision aid or associated documentation…

1. Shows the negative and positive features of options with equal detail (e.g., using similar fonts, sequence, presentation of statistical information)
2. Provides citation to the evidence selected
3. Provides a production or publication date
4. Provides information about the update policy
5. Provides information about the levels of undertainty around event or outcome probabilities (e.g., by giving a range or by using phrases such as “our best estimate is…”)
6. Provides information about the funding source used for development
7. **Checklist to Assess Acceptability of Coaching**

**Five items from Decision Support Assessment Tool (DSAT)**[^23^](file:///C:\Users\lstupar\Dropbox\ACTIVE%20%20UCSF\20190604%20DOD%20Manuscript%20Resubmission\20190418%20UCSF%20DOD%20PACE%20Pilot%20Table%202.docx#_ENREF_23)

Did the coach…

1. Discuss the patient’s knowledge of his clinical condition, options, and outcomes? [Y/N]
2. Discuss with the patient what is most important? [Y/N]
3. Discuss the patient’s preferred role in decision making and acknowledge who else is involved in making the decision? [Y/N]
4. Discuss strategies to involve others (e.g. ask questions, share what is important)? [Y/N]
5. Facilitate access to sources of factual information about the clinical condition, options, and outcomes? [Y/N]
